# Descemet’s membrane endothelial keratoplasty in an eye with iridocorneal endothelial syndrome and rare association of corneal ectasia

**DOI:** 10.1177/25158414251343968

**Published:** 2025-08-18

**Authors:** Mohammad Saleki, Preston Lee, Caroline Thaung, Zahra Ashena

**Affiliations:** Queen’s Hospital, Romford, London, UK; Queen’s Hospital, Romford, London, UK; Department of Medicine, Queen Mary University of London, London, UK; Department of Eye Pathology, University College London, London, UK; Queen’s Hospital Romford, Rom Valley Way, Romford, RM7 0AG, UK

**Keywords:** corneal dystrophy, DMEK, endothelial keratoplasty, ICE syndrome, keratoconus

## Abstract

We report the first case of concurrent iridocorneal endothelial (ICE) syndrome and keratoconus, treated successfully with Descemet’s membrane endothelial keratoplasty (DMEK). A 60-year-old male presented with gradual visual deterioration in his left eye over 4 years. Best corrected visual acuity was 1.1 LogMar, with corneal stromal oedema. Hypertonic saline and systemic acyclovir provided no improvement. Further examination revealed peripheral anterior synechiae and possible ICE syndrome. Combined cataract surgery and adapted DMEK were performed, using right eye data for intraocular lens calculation. Postoperative histopathology confirmed ICE syndrome. Two months postoperatively, vision improved to 0.54 LogMar, with normal intraocular pressure and optical coherence tomography. Ten months later, unaided visual acuity reached 0.4 LogMar, with no significant changes observed in regular follow-ups. The patient remains satisfied with his vision. This case highlights the rare association of keratoconus with Chandler Syndrome and the first report of such a case where DMEK was used as management. The diagnosis of ICE syndrome complicates treatment, however, despite the challenges, DMEK demonstrated promising results for ICE-related corneal oedema in a patient with concurrent keratoconus, offering improved visual acuity and no complications.

## Introduction

Iridocorneal endothelial (ICE) syndrome is a rare ocular disorder characterised by the abnormal proliferation and migration of corneal endothelial cells to the iris and iridocorneal angle. This migration leads to various clinical manifestations, including secondary angle-closure glaucoma, corneal oedema and iris atrophy.^1–4^ ICE syndrome primarily affects women in their fourth and fifth decades of life and is typically unilateral in nature.^5^

The hallmark of ICE syndrome lies in the transformation of single-layered hexagonal endothelial cells into abnormal ‘ICE cells’. These cells exhibit epithelial characteristics, being larger in size, rounded, potentially multi-layered and possessing desmosomes and microvilli. There are three recognised subtypes of ICE syndrome: Cogan Reese Syndrome,^1^ Chandler Syndrome, the most common among Caucasians^5,6^ and Essential Iris Atrophy, which is more prevalent in pigmented eyes.^2,7,8^

Keratoconus (KC), on the other hand, is a progressive corneal ectatic disorder characterised by thinning and steepening of the cornea, leading to visual impairment.^9^ This condition usually presents in the second decade of life. Its association with ICE syndrome is exceptionally rare with only few reported cases.^1,6–8,10–12^ In this case report, we present a unique instance of the concomitant occurrence of ICE syndrome and keratoconus. Through this report, we aim to provide insights into the clinical presentation, management and outcome of this rare association, giving insight on potential diagnostic and therapeutic strategies for similar cases in the future. To the author’s knowledge, this is also the first reported case where Descemet’s membrane endothelial keratoplasty (DMEK) was used in the management of ICE syndrome with KC.

## Case presentation

**Figure 1.**
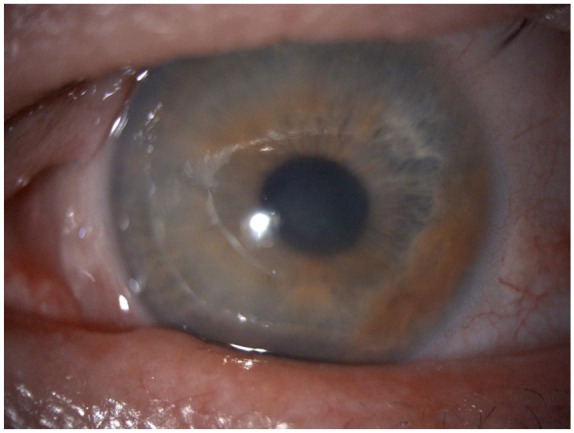
Preoperative corneal oedema of the left eye.

A 60-year-old Caucasian male with an otherwise clear past ophthalmic history presented with gradual, painless deterioration of vision in his left eye over 4 years. He had no history of atopic disease, eczema or eye rubbing. His uncorrected visual acuity was 1.1 LogMar in his left eye and 0.1 LogMar in the right eye. On examination, the left cornea showed stromal oedema (Figure 1) with a central thickness of 765 µm. There was no evidence of ocular inflammation, keratic precipitates, corneal scarring or neovascularisation in the affected eye, while the fellow eye appeared pristine with no endothelial dystrophy. Due to corneal oedema, specular microscopy of the left eye was inconclusive. However, in the right eye, the endothelial cell count and features were within the normal range.

**Figure 2.**
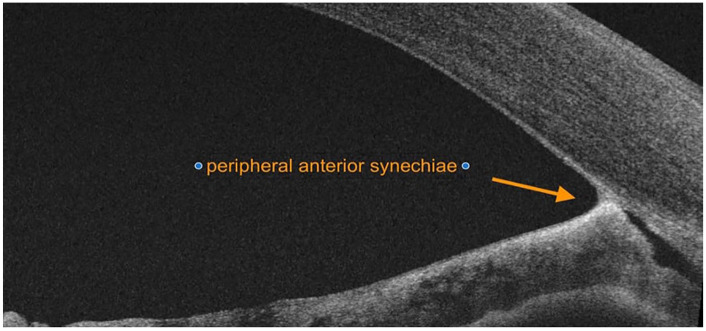
Peripheral anterior synechiae on optical coherence tomography.

He was started on sodium chloride 5% eye drops 4 times daily. Despite the absence of compelling evidence indicating a herpetic infection in his medical history or clinical examination, the high prevalence of herpes simplex virus (HSV) warranted the start of empirical systemic acyclovir treatment at a dosage of 400 mg 5 times daily for 2 weeks, yielding no significant improvement. However, during examination, an area of raised, mildly pigmented iris with iridocorneal contact was noted in the far-periphery of the inferotemporal iris (Figure 1). On further examination, optical coherence tomography (OCT) revealed anterior synechia peripherally between 3 and 5 o’clock (Figure 2). Oncology consultation excluded uveal malignancies.

Given the suspected diagnosis of ICE syndrome, combined cataract surgery and DMEK was planned. Owing to corneal oedema, auto-refraction and biometry tests for the left eye were inconclusive, therefore, the right eye’s keratometry data were used for left eye intraocular lens (IOL) calculation.

**Figure 3.**
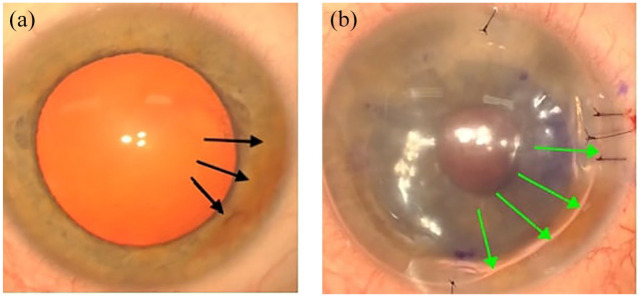
Presence of anterior synechia pre (a) and post (b) injection of gas. Black arrows highlight anterior synechia and green arrows show the gas margin.

The left eye underwent combined phacoemulsification and DMEK with aqueous sampling to exclude HSV, Varicella zoster virus (VZV) and cytomegalovirus (CMV). The main incision was made 1 clock hour above the synechia. Following uneventful extraction of cataract and insertion of a hydrophobic IOL, a fairly small 7.5 mm DMEK was planned to avoid the peripheral anterior synechiae (PAS). The donor Descemet tissue was injected to the anterior chamber and unfolded and centred after shallowing the chamber. The donor tissue was secured against the host stroma using 20% sulphur hexafluoride gas. The main incision and paracentesis were closed using 10-0 nylon sutures, followed by subconjunctival injection of Cefuroxime (Zinacef^®^ 250 mg; Sandoz Limited, Bracknell, UK) and Dexamethasone. Post-operatively, the patient was advised on 48 h of supine posturing, during which he was allowed 5 min break per hour. He was also started on Acetazolamide slow release 250 mg twice daily for 2 days, Cyclopentolate hydrochloride 1% eye drops 3 times a day for 1 week, Ofloxacin 0.3% eye drops 4 times daily for 2 weeks and Dexamethasone phosphate 0.1% preservative free eye drops 6 times daily with a slow tapering regime over 4 months, followed by once daily for 2 years. Figure 3 shows anterior synechia pre (Figure 3(a)) and post (Figure 3(b)) injection of gas.

**Figure 4.**
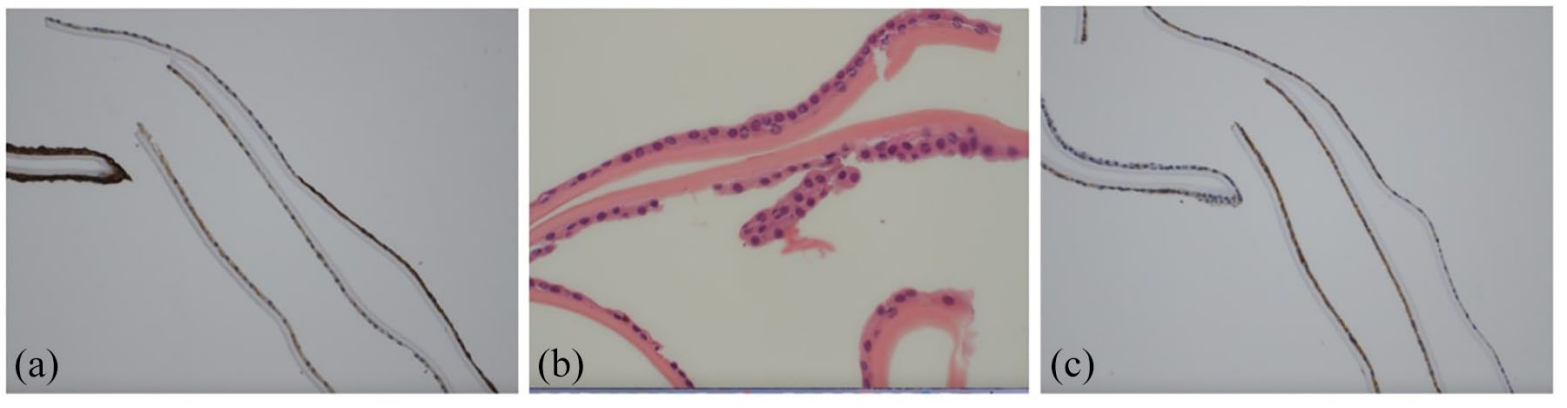
Histological images of excised tissues, showing a mixed expression of epithelial and endothelial markers. (a) Expression of epithelial markers AE1/AE3; (b) bilayering of cells, characteristic of epithelial cells; (c) expression of endothelial marker CD56.

The excised Descemet membrane was sent for histopathology studies. Microscopy of the excised Descemet membrane revealed a covering of low cuboidal cells, mainly in a monolayer with occasional bi- or multilayering. Cell density appeared higher than in typical DMEK specimens. Immunohistochemistry for epithelial markers (AE1/AE3, CK8/18) and endothelial markers (CD56, vimentin) demonstrated a mixed expression pattern in morphologically similar cells, supporting metaplasia rather than epithelial downgrowth. These findings were consistent with ICE syndrome. Figure 4 provides representative images.

Following surgery, the corneal oedema gradually settled, the corneal stitches were removed at 2 weeks post-operatively, the patient achieved an unaided visual acuity (UAVA) of 0.54 LogMar 2 months postoperatively, with a clear cornea, normal optic disc and normal macular OCT findings. The intraocular pressure (IOP) was 14 mmHg in the right eye and 16 mmHg in the left eye, with a cup-to-disc ratio of 0.55 bilaterally. Humphrey Visual Field test did not show a convincing defect. Subjective refraction in the left eye measured +2.00 D −2.5 D 180° with no significant improvement in the best-corrected visual acuity beyond 0.5 LogMar. Despite the patient’s satisfaction with his vision and denial of any history of amblyopia, there was no justification for his partially improved visual acuity.

**Figure 5.**
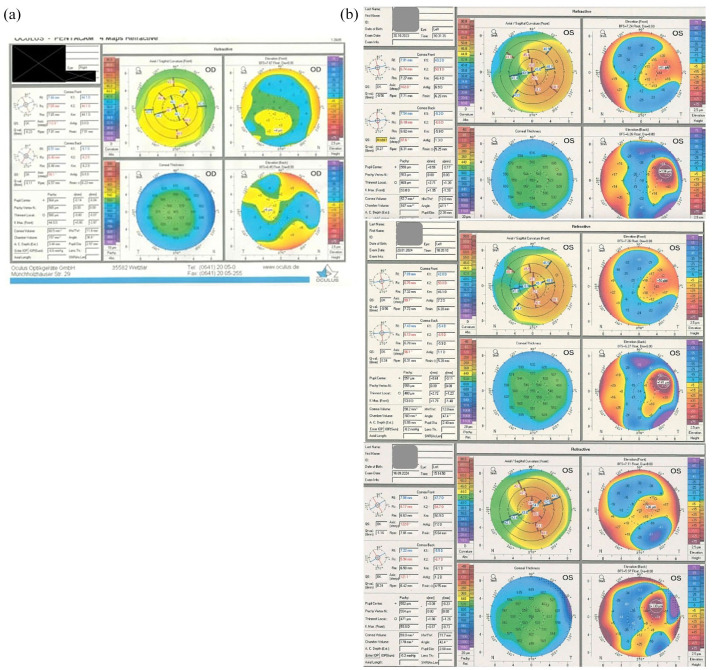
Pentacam^®^ HR results for right eye (a) showing normal findings and left eye (b) demonstrating infero-temporal ectasia with reduced thickness profile.

At this stage, corneal tomography was performed, using Pentacam^®^ HR Device (Figure 5). His keratometry parameters showed a significant difference between the eyes, with a maximum keratometry (Kmax) of 45.2 D in the right eye and 54.9 D in the affected left eye, and thinnest points of 556 and 482 µm, respectively. The axial map demonstrated an infero-temporal steepening, which corresponded with the posterior elevation map. Considering the infero-temporal anterior synechia, it was presumed that an adjacent pseudo-ectasia may have occurred secondary to the localised synechia. However, taking into account the corneal thickness map, keratoconus was the presumed condition, which had not been diagnosed previously.

Ten months postoperatively, the patient’s UAVA in the left eye improved to 0.4 LogMar, with pinhole improvement to 0.1 LogMar. He expressed contentment with his vision and declined further trials with glasses or rigid contact lenses.

Regular Pentacam and specular microscopy showed no significant changes to the left eye, up until 18 months post-surgery. However, his latest Pentacam scan showed progressing ectasia (Figure 5(b)), for which corneal crosslinking was offered.

His right eye remains normal with a UAVA of 0.02 LogMar, pristine cornea and normal IOP. The patient continues to attend regular cornea and glaucoma appointments.

## Discussion

We report the first case of concurrent KC and ICE syndrome, who underwent DMEK to manage their corneal decompensation. Also, our patient is the most senior patient with keratoconus and ICE syndrome, amongst the few reported cases in the literature. While the corneal topography does not typically indicate keratoconus, it does suggest the presence of an ectatic disorder of the cornea that exhibits progressive characteristics.

Keratoconus is mostly associated with atopic diseases,^13^ eye rubbing^14^ and intellectual disabilities,^15^ and rarely reported in the presence of corneal dystrophies. Concurrence of KC and ICE syndrome is very rare with only a few published reports.^1,6–8,10–12^ The pathogenesis remains unclear; however, it has been suggested since the posterior layers of the cornea, and the iris stroma have a common embryological origin, the combination of these two conditions may have an inter-related pathogenesis.^16^

The specific subtype of ICE syndrome can be diagnosed based on varying features. Chandler Syndrome typically presents with unilateral worsening vision, corneal oedema, normal IOP and epithelial bullae.^17^ Whereas Cogan-Reese Syndrome is characterised by proliferation of corneal endothelium into the anterior chamber angle and iris. Essential iris atrophy, however, as per the name is characterised by iris atrophy, which often leads to hole formation.^18^ This report therefore highlights the infrequent association of KC with Chandler Syndrome within the domain of ICE Syndrome. There have been four other instances of unilateral KC with ICE syndrome.^6,10,12,19^ Subsequent cases demonstrated bilateral KC with ICE syndrome.^1,17,19^ The main presenting complaint in the majority of the reported cases has been progressive vision loss secondary to keratoconus, for which disease stabilisation with corneal crosslinking was offered where needed,^10^ along with visual rehabilitation, which ranged from hard contact lenses^1,7,8^ in mild to moderate keratoconus to full thickness keratoplasty^1,7,19^ in advanced disease with apical scarring. There was also one case of induced KC following globe massage in a patient with ICE syndrome, however this was not an existing association.^19^ Our patient was the only patient, who developed corneal oedema, which was in fact the first manifestation of the disease. The difference in details of the reported cases is laid out in Table 1.

**Table 1.** Summary of all cases of concurrent ICE syndrome and keratoconus reported in literature.

Presented cases	Age	Main issues	Sex	KC	ICE (subtype)	Specular microscopy	Corneal decompensation/oedema	Management
Current case	60	Corneal stromal oedema and peripheral anterior synechiae	Male	Unilateral	Unilateral Chandler Syndrome	Inconclusive	Present	DMEK
Phylactou et al.^10^	30	Corectopia progressive inferior corneal ectasia	Male	Unilateral	Unilateral (type unspecified)	Changes of ICE (unspecified)	Absent	Epithelium-off accelerated CXL
Kalantan^6^	34	Advanced KC with apical scar and corectopia	Female	Unilateral	Unilateral Chandler Syndrome	Decreased endothelial count, rounding of endothelial cells angles and eccentric dark areas	Absent	Penetrating keratoplasty, pupilloplasty
Li et al.^11^	43	Progressive decrease of vision secondary to keratoconus and mild corneal haze	Male	Bilateral	Unilateral Chandler Syndrome	Light-dark reversal pattern, pleomorphism	Absent	Conservative, 0.3% Hypromellose lubricating eye drops, 2% sodium cromoglycate eye drops and oral antihistamines
Gus et al.^12^	47	Corneal thinning with anterior protrusion, central leucoma, iris atrophy	Female	Unilateral	Unilateral Essential Iris Atrophy	Not stated	Absent	Not stated
De Maria et al.^7^	38	Progressive unilateral decreased vision due to advanced ectasia	Male	Bilateral	Unilateral essential iris atrophy	Abnormal endothelial morphology	Absent	Rigid gas permeable contact lens and glaucoma drainage device implantation
Chakrabarty^1^	32	Gradual reduction of vision owing to bilateral advanced keratoconus and right glaucoma	Male	Bilateral	Unilateral Cogan-Reese Syndrome	Pleomorphism, polymegathism, light-dark reversal and a low cell density	Absent	Rigid gas permeable contact lens, topical antiglaucoma
Blair et al.^8^	47	KC and glaucoma	Female	Bilateral	Bilateral essential iris atrophy, posterior polymorphous dystrophy	Changes of ICE and posterior polymorphous dystrophy	Absent	Initially beta-blocker shifted to neptazane after development of wheeze, RGPCL

CXL, corneal collagen cross-linking; DMEK, Descemet’s membrane endothelial keratoplasty; ICE, iridocorneal endothelial; KC, keratoconus.

Due to the rarity of the concurrent KC and ICE syndrome, the diagnosis and identification of appropriate treatment options may be challenging. The asymptomatic nature of early-stage KC means that many cases remain undiagnosed unless assessed by corneal tomography. We believe our patient’s ectasia has been a longstanding condition which went undiagnosed throughout his life before his presentation with ICE syndrome. This is because he was delighted with an UAVA of 0.4 LogMar post-op, feeling he was having his ‘normal’ vision. This case, thus, displays the importance of extensive examination in practice to uncover such diagnoses. In accordance with the theory of an underlying viral infection with HSV leading to low-grade inflammation at the corneal endothelial level, resulting in its unusual epithelial-like activity or ICE syndrome, and the prevalence of HSV in the West, aciclovir was initiated. This decision was made despite the absence of any history of previous HSV keratitis or signs of neovascularisation/sectoral neurotrophia, which did not yield any improvement. A negative aqueous fluid PCR test for HSV, VZV and CMV infections excluded the role of viruses in the development of his condition.

In Kalantan’s case, penetrating keratoplasty (PK) was used to rectify the unilateral advanced KC and ICE syndrome. While PK could potentially address both KC and the abnormal endothelium, it was avoided in our case, as corneal decompensation was the consequence of endothelial failure, hence endothelial keratoplasty would be the procedure of choice. Prognosis of PK in ICE syndrome has been relatively poor with higher rates of rejection and late endothelial failure, necessitating multiple keratoplasties.^20,21^ On the other hand, DMEK has previously been shown to have a 0.7% rejection rate,^22^ with rapid visual recovery, better maintenance of ocular integrity, and avoidance of suture-related complications.^23,24^

It is known that DMEK in complex anterior segment pathologies is associated with higher re-bubbling rates and consequently higher graft failure.^25^ A meta-analysis has shown that using 20% SF6 and post-op supine posture is associated with a lower rate of graft detachment and rebubbling in DMEK procedure,^26^ compared to air tamponade. We used 20% SF6 for graft tamponade and our patient was compliant with maintaining a supine posture for 2 days post-operatively. He did not develop a graft detachment hence no rebubbling was required. Short-term and long-term studies of eyes with ICE syndrome treated with DMEK highlighted no graft failure or rejection at 6 and 36 months.^27,28^ A 2018 case series involving four eyes with ICE revealed a significant improvement in best-corrected visual acuity at both 6- and 24-month post-procedure.^26^ DMEK and Descemet Stripping Automated Endothelial Keratoplasty (DSAEK) have been associated with improved outcomes in patients with endothelial decompensation and anterior segment diseases. Between the two, DSAEK is technically less challenging procedure, however, DMEK has demonstrated superior visual outcomes compared to DSAEK.^22^

To achieve the best outcome on the rare occasion of DMEK in ICE syndrome, the surgery was performed by a consultant ophthalmologist, with the experience of several DMEK procedures before. Also, to avoid the anterior synechia interfering with unfolding of the graft, a slightly smaller graft of 7.5 mm was used. This is also highlighted by previous studies that graft size in ICE syndrome should not be based on the recipient’s corneal diameter but the extent of PAS.^28^ In spite of the merits of endothelial keratoplasty procedures, in patients with ICE syndrome, these do not completely excise the abnormal endothelium and therefore are not able to decisively halt the progression of PAS and glaucoma.^6^ As such, owing to the presence of both a mildly raised iris and PAS, the utilisation of cataract surgery would aid in flattening the iris as well as widening the angle and reducing the occurrence of angle closure. Synechiolysis is usually attempted by glaucoma specialists to manage the secondary angle closure in ICE syndrome.^29–31^ This was avoided in our patient since he had only a localised PAS, and the rest of his irido-corneal angle was open. Moreover, the release of fibrin and blood to the anterior chamber following synechiolysis can significantly interfere with the graft attachment.^32^

The often relatively shallow anterior chamber in the ICE syndrome,^33,34^ helps with the easy unfolding of the graft, however, the anterior chamber was deep in our case (5.55 mm), due to the concurrent keratoconus. This was addressed by releasing the aqueous fluid from the paracentesis, which shallowed the anterior chamber and eased the unfolding manoeuvre.

Although clear management pathways for this combination of diseases are lacking, the treatment options discussed offer a valuable overview of how addressing individual symptoms can yield optimal outcomes. Evident in this case, DMEK demonstrates an exceptional prognosis for patients with ICE and keratoconus, notably with ICE-related corneal oedema. In Kalantan’s case, PK yielded excellent results, improving the patient’s UAVA from counting fingers at 2 feet to 20/60 two years post-op.^6^ Although short-term prognosis after DMEK in ICE syndrome is favourable, the long-term outcomes are guarded given the nature of progression of PAS and secondary glaucoma.^35,36^ This should be considered, given rarity of ICE syndrome and limited literature, with the longest reported outcomes being 3 years.^28^

Epithelium-off accelerated corneal collagen cross-linking (CXL) has shown to also halt KC progression for a case presented in a 30-year-old male.^10^ CXL was not used in the treatment of KC in other reported patients with ICE syndrome given ICE syndrome appears in adulthood when KC progression is less probable.^28^

In conclusion, the ambiguous nature of ICE syndrome concomitant with KC can fundamentally make the medical management of this condition very challenging. This highlights the importance of preoperative planning to cater for unique considerations. Endothelial Keratoplasty still ultimately offers a potential pathway forward for patients with this condition as it offers swift visual recovery with minimal refractive alterations, eliminates the need for sutures, and more effectively preserves the integrity and innervation of the corneal recipient.^37^ Based on our experience, the below tips were helpful to achieve a successful outcome:

- The surgery is to be carried out by an experienced surgeon.
- The DMEK size is to be adjusted by the extension of PAS.
- There should be no attempt at synechiolysis prior to DMEK.
- Use of 20% SF6 gas and post-op supine posture for a better tamponade is encouraged.

Additionally, it is also pertinent to be aware of the age of the patient in this case, highlighting the importance of considering ICE syndrome and KC in older adults with similar presentations.

## Declarations

**Ethics approval and consent to participate:** Our institution does not require ethical approval for reporting individual cases, however written consent was obtained from the patient to publish this case.

**Consent for publication:** Written informed consent to publish the case report and related data was obtained.

**Availability of data and materials:** Not applicable.
